# Supplementary material for: Comparative Phytochemical Analyses of Flowers from Primula veris subsp. veris Growing Wild and from Ex Situ Cultivation in Greece
Source: Foods. 2023 Jul 6;12(13):2623. doi: 10.3390/foods12132623 (PMC10341331; doi:10.3390/foods12132623)
Supplement: Supplementary file 1 [file foods-12-02623-s001.zip › foods-2491217-supplementary.pdf]

foods

MDPI

SUPPLEMENTARY FILE

Comparative phytochemical analyses of flowers from *Primula veris* subsp. *veris* growing wild and from *ex situ* cultivation in Greece

Konstantia Graikou[1], Anna Mpishinioti[1], Nikolaos Tsafantakis[1], Eleni Maloupa[2], Katerina Grigoriadou[2] and Ioanna Chinou[1,*]

[1] Laboratory of Pharmacognosy and Chemistry of Natural Products, Faculty of Pharmacy, National and Kapodistrian University of Athens, Panepistimiopolis, Zografou, 15771, Athens, Greece.
[2] Balkan Botanic Garden of Kroussia-Laboratory for the Conservation and Evaluation of Native and Floricultural Species, Institute of Plant Breeding and Genetic Resources, Hellenic Agricultural Organization-DIMITRA, Thermi, P.O. Box 60458, GR-570 01 Thessaloniki, Greece.
* Correspondence: ichinou@pharm.uoa.gr

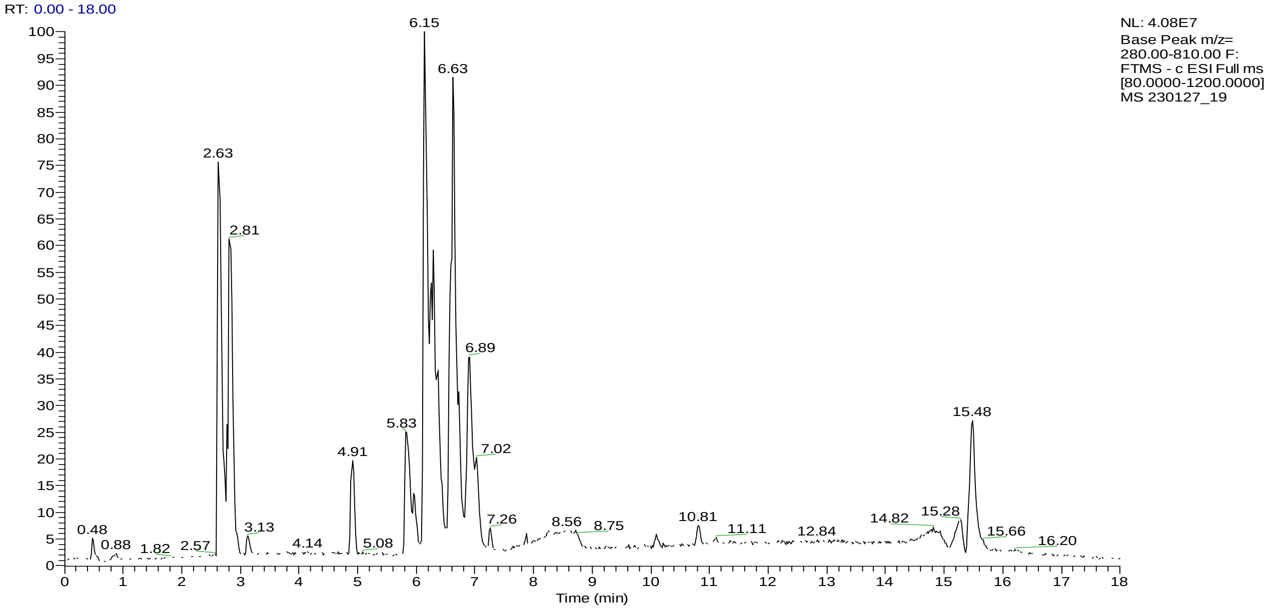

Figure S1. LC chromatogram of the wild *Primula veris* subsp. *veris* from Lake Prespa National Park by UHPLC-HRMS analysis

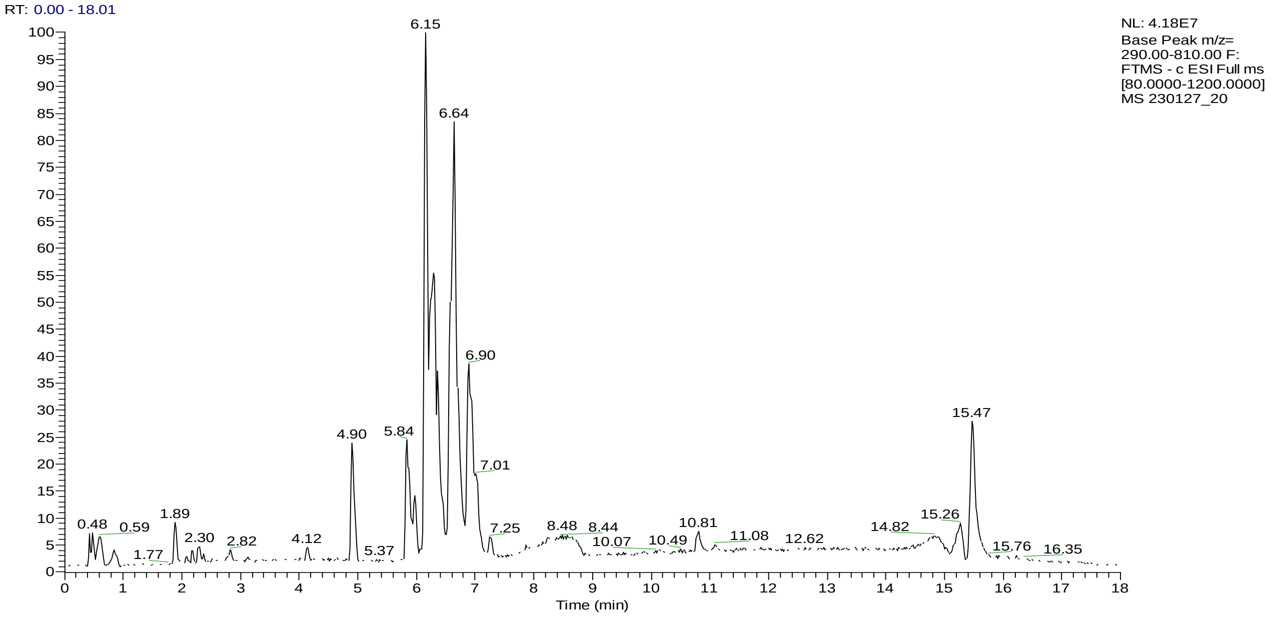

Figure S2. LC chromatogram of the *ex situ* cultivated *Primula veris* subsp. *veris* by UHPLC-HRMS analysis

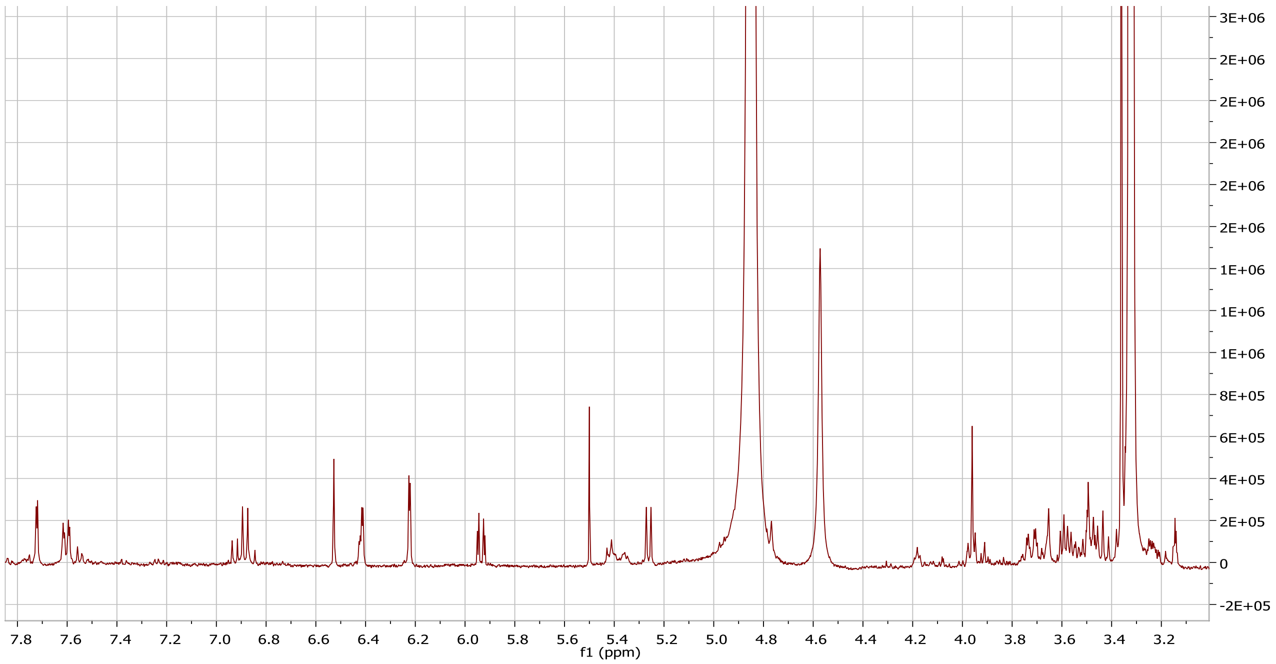

Figure S3. ^{1}H-NMR spectrum of compounds 1 and 2 (CD3OD, 400 MHz).

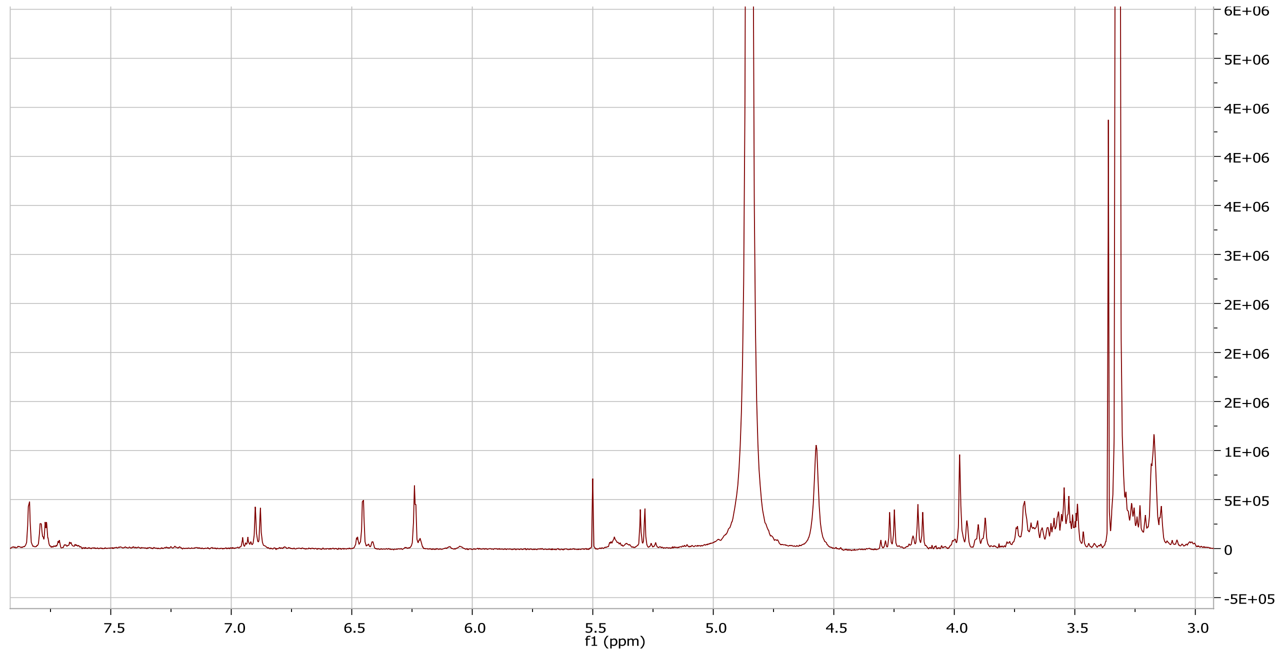

Figure S4. ^{1}H-NMR spectrum of compounds 3 and 4 (CD3OD, 400 MHz).

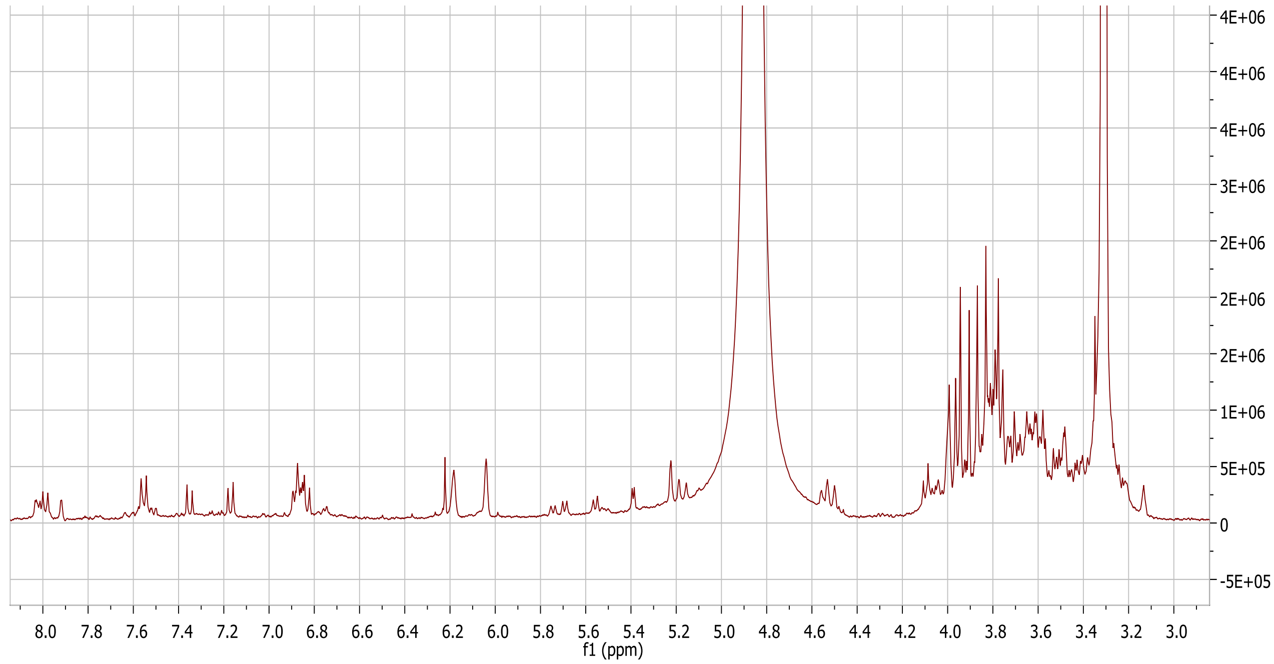

Figure S5. ^{1}H-NMR spectrum of compounds 5 and 6 (CD3OD, 400 MHz).

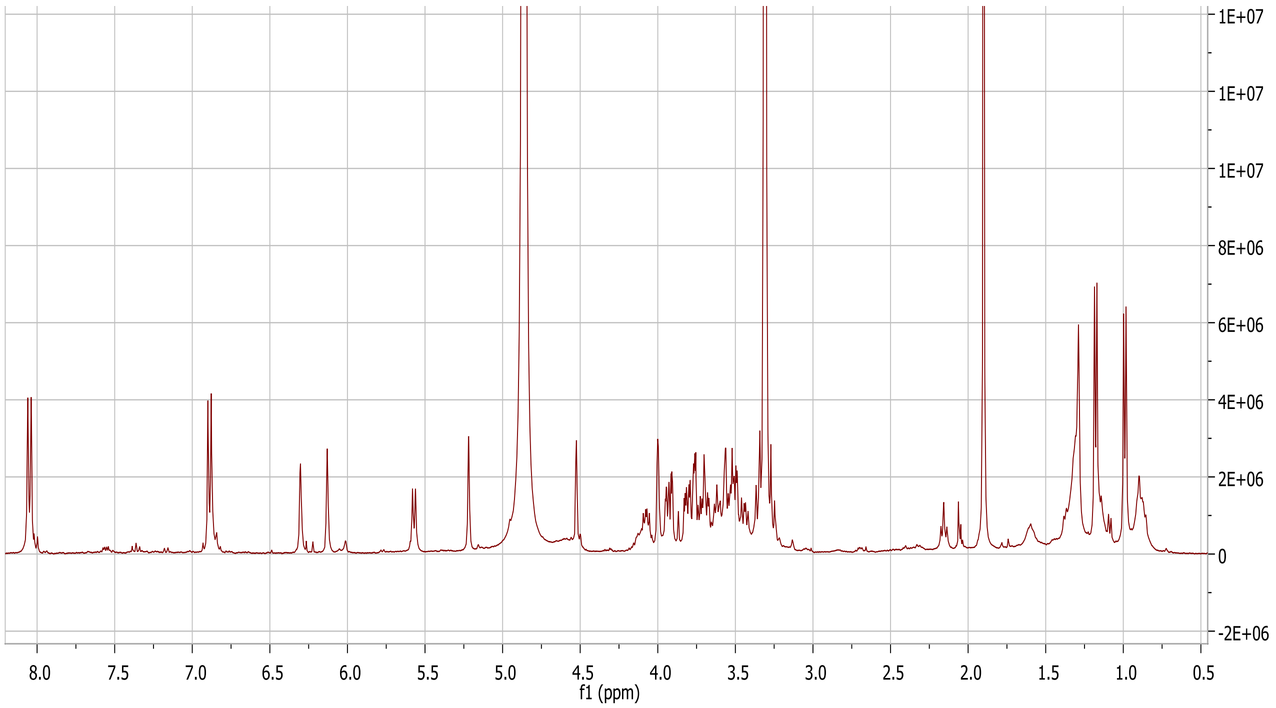

Figure S6. ^{1}H-NMR spectrum of compound 7 (CD3OD, 400 MHz).

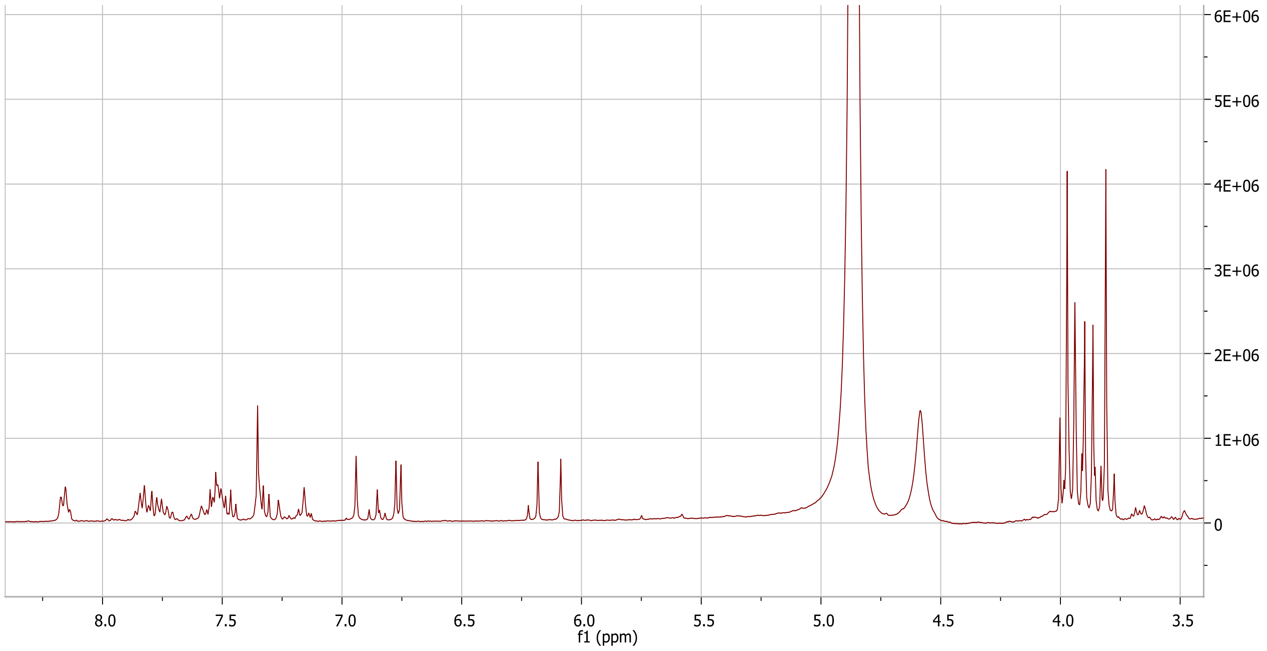

Figure S7. ^{1}H-NMR spectrum of compounds 7, 8 and 9 (CD3OD, 400 MHz).

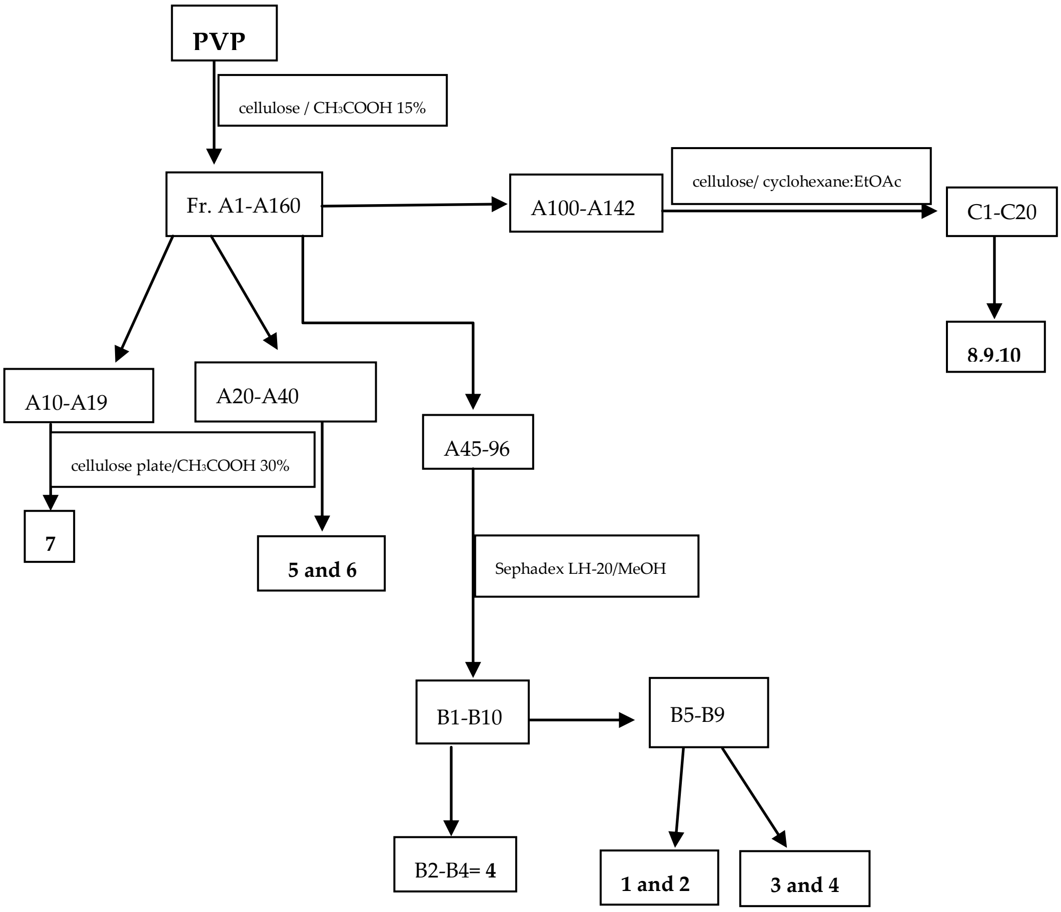

Figure S8. Fractionation and purification flow chart of the infusion of wild *Primula veris* subsp. *veris*
